# The Synthetic Cannabinoid URB447 Exerts Antitumor and Antimetastatic Effect in Melanoma and Colon Cancer

**DOI:** 10.3390/ph15101166

**Published:** 2022-09-20

**Authors:** Aitor Benedicto, Beatriz Arteta, Andrea Duranti, Daniel Alonso-Alconada

**Affiliations:** 1 Department of Cell Biology and Histology, Faculty of Medicine and Nursery, University of the Basque Country, 48940 Leioa, Bizkaia, Spain; 2 Department of Biomolecular Sciences, University of Urbino Carlo Bo, 61029 Urbino, Italy

**Keywords:** liver metastasis, cannabinoids, URB447, colon cancer, melanoma

## Abstract

The endocannabinoid system is widespread through the body and carries out a wide variety of functions. However, its involvement in other pathologies, such as cancer, still needs further attention. We aim to investigate the role of CB2 receptor during melanoma and colorectal cancer (CRC) aggressiveness and metastatic growth in the liver. We used the synthetic cannabinoid URB447, a known CB2 agonist and CB1 antagonist drug, and studied prometastatic ability of mouse B16 melanoma and MCA38 CRC cells, by means of proliferation, apoptosis, cell cycle, migration and matrix degradation in vitro upon URB447 treatment. We reported a dose-dependent viability decrease in both tumor types. This result is partly mediated by apoptotic cell death and cell cycle arrest in G1/G0 phase, as observed through flow cytometry. Melanoma and CRC cell migration was affected in a dose-dependent fashion as observed through scratch assay, whereas the secretion of matrix degrading proteins metalloprotease 2 (MMP2) and 9 (MMP9) in tumor cells did not significantly change. Moreover, daily treatment of tumor bearing mice with URB447 decreased the development of liver metastasis in a melanoma model in vivo. This proof of concept study points out to the synthetic cannabinoid URB447 as a potential candidate for deeper studies to confirm its potential as antitumor therapy and liver metastasis treatment for CRC and melanoma.

## 1. Introduction

The endogenous cannabinoid system (ECS) represents a ubiquitous neuromodulatory system in the body, with a wide spectrum of different functions. It is composed by the cannabinoid receptors and their endogenous ligands and the enzymes that synthesize and degrade these ligands [1]. Cannabinoid receptor 1 (CB1) and cannabinoid receptor 2 (CB2) are the main effectors of the ECS functions, along with other receptors such as such as PPAR’s and Transient Receptor Potential (TRP) channels [2]. CB1 and CB2 receptors are G protein coupled receptors that exert several intracellular changes and, therefore, mediate cellular processes such as gene transcription, cell motility and angiogenesis [3,4].

ECS receptors are widespread throughout the body and involved in different physiological processes. It is the omnipresence what makes CB receptors a potential target for several pathologies [5,6]. In fact, CB receptors are expressed in a wide variety of cancers with different origin, such as glioma and melanoma, to name a few [7,8]. However, the role of these receptors is not yet very well understood. While in some cancers, such as glioma or melanoma, the activation of CB1 and CB2 lead to impaired protumoral activities [9,10], in other malignancies such as non-small cell lung carcinoma, these receptors act as protumoral mediators [11].

To date, several studies have proven the effectiveness of some cannabinoids against cancer. Cannabidiol (CBD) is the main non-psychoactive constituent of Cannabis. CBD has shown anticancer properties, affecting diverse tumoral processes. CBD blocked the cell cycle of gastric cancer cells, leading to reduced CDK2/Cyclin E protein levels [12] and exerted proapoptotic effect in breast cancer cells through endoplasmic reticulum stress, driving to apoptosis [13]. Intriguingly, CBD showed an antagonistic effect to CB1 while having an agonistic effect to CB2 [14]. 

The in vitro anticancer properties of cannabinoids include cell cycle arrest, the induction of cancer cell apoptosis, impaired migration and invasion, reduction of matrix metalloprotease 2 and 9 (MMP-2,9), impairment of angiogenic response through VEGF downregulation, inhibition of epithelial to mesenchymal transition (EMT) and lead to affected metastatic growth [15,16].

However, a short number of studies has validated this in vitro effect using animal models. In fact, just one study has tested in vivo the effect of a cannabinoid, specifically a hexahydrocannabinol analog, on colorectal cancer (CRC) metastasis in a xenograft model of CRC [17]). Regarding to melanoma, only six studies have validated the efficacy of cannabinoids during cancer development in vivo [18]. Despite the animal models studied, none of them analyzed the effect of cannabinoids during the metastatic process in the liver. This organ represents a common target organ for several cancers, such as CRC, pancreatic cancer and melanoma [19], which significantly complicates the prognosis and survival of patients. It is interesting to note that several cannabinoids have a high impact on the development of a wide array of liver pathologies. 

Based on the above-mentioned evidences, stimulating CB2 receptor while antagonizing CB1 receptor may represent a promising therapeutic option for the treatment of CRC and melanoma liver metastasis. 

**Figure 1:**
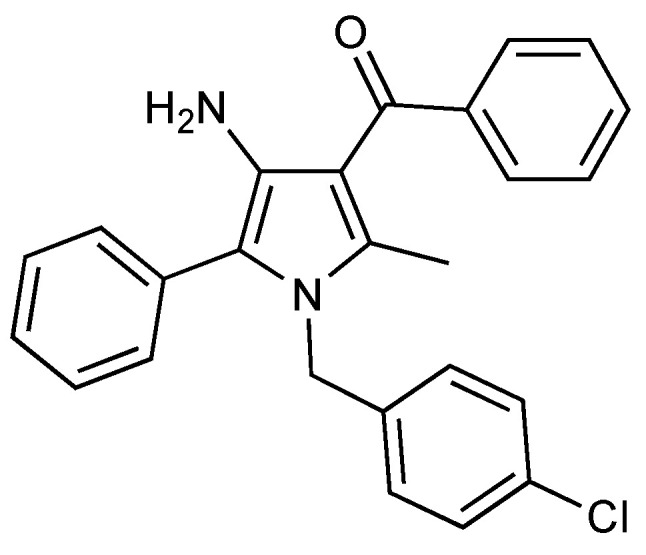
Chemical structure of the synthetic cannabinoid URB447.

URB447 ({[4-amino-1-(4-chlorobenzyl)-2-methyl-5-phenyl-1H-pyrrole-3-yl](phenyl) methanone}) (Figure 1) is a synthetic cannabinoid ligand able to act as CB2 agonist and CB1 antagonist. Its ability to show anorexiant activity without the typical side effects of drugs acting on the central nervous system was originally discovered, due to the fact the molecule reduces feeding and body-weight gain in mice with a peripherally restricted action [20]. More recently, it has been demonstrated that URB447 reduces brain injury and the associated white matter demyelination after hypoxia-ischemia in neonatal rats [21].

Therefore, we tested the anticancer efficacy of the synthetic cannabinoid URB447 in CRC and melanoma in vitro and further explored its function using a syngeneic model of liver metastasis in vivo. It is tempting to hypothesize that the ability of URB447 to exert a stimulatory effect in CB2 may reduce the protumoral activity of both melanoma and CRC in vitro and prevent metastatic growth in the liver.

## 2. Results

### 2.1. Antitumor Effect of URB447 in Cancer Cell Viability

**Figure 2:**
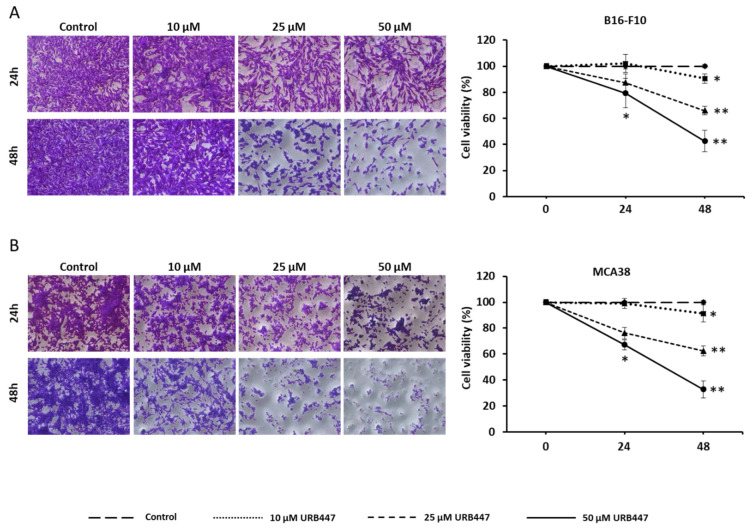
Tumor cell viability upon treatment with URB447. Melanoma (**A**) and CRC cancer cells (**B**) were incubated in the presence of URB447 for 24 and 48 h. Cell viability was measured using Prestoblue™ viability assay. Images show representative cell population at the time of the measurement (*n* = 3). Differences were considered statistically significant for * *p* < 0.05, ** *p* < 0.01 using Student *t* test.

We have checked the potential of URB447 to modulate melanoma and CRC cell viability. Our results show that this synthetic cannabinoid interferes with tumor cell viability in both cancer models. In detail, URB447 10 µM did not affect cell viability after 24 h, whereas 25 µM and 50 µM exerted an antitumor effect at this time point. However, 10 µM significantly reduced cancer cell viability about 10% after 48 h in B16-F10 melanoma cells, whereas 25 µM and 50 µM drove to 40% and 60% cell death, respectively (Figure 2A). Regarding MCA38, the same trend was observed after 48 h, reducing cell viability 10%, 40% and 67% when treated with 10 µM, 25 µM and 50 µM, respectively (Figure 2B). 

### 2.2. Apoptotic Effect of URB447 in Cancer Cells

**Figure 3:**
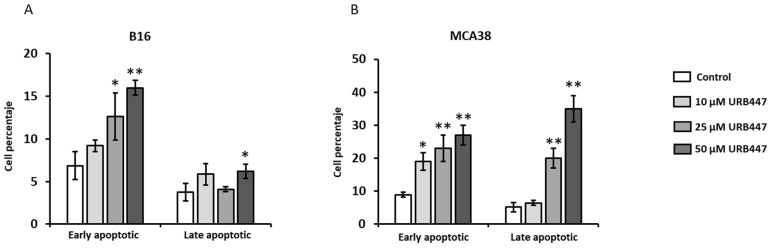
Apoptotic effect of URB447. Melanoma (**A**) and CRC cancer cells (**B**) were incubated in the presence of URB447 for 24 h. Flow cytometry was carried out to analyze early and late apoptotic cells. Images show representative FACS result (*n* = 3). Differences were considered statistically significant for * *p* < 0.05, ** *p* < 0.01 using Student *t* test.

To uncover the mechanisms for reduced viability, we analyzed the effect of URB447 in the apoptotic cell death in both melanoma and CRC models. As shown in Figure 3, URB447 promotes apoptosis in both models. URB447 promoted apoptotic cell death in B16-F10 melanoma cells in a dose-dependent manner after 24 h. In detail, whereas 10 µM and 25 µM slightly increased apoptotic cells, 50 µM rendered melanoma cells to apoptosis mediated cell death, increasing 3-fold early apoptotic cell counts (Figure 3A). Regarding MCA38, the same proapoptotic pattern was observed in cells treated with 10 µM, 25 µM and 50 µM URB447. In this regard, 10 µM increased 2,5-fold the percentage of apoptotic cells, whereas 25 and 50 µM led to 4-fold augmentation in early apoptotic cell numbers (Figure 3B).

### 2.3. Cell Cycle Interference upon URB447 Treatment

**Figure 4:**
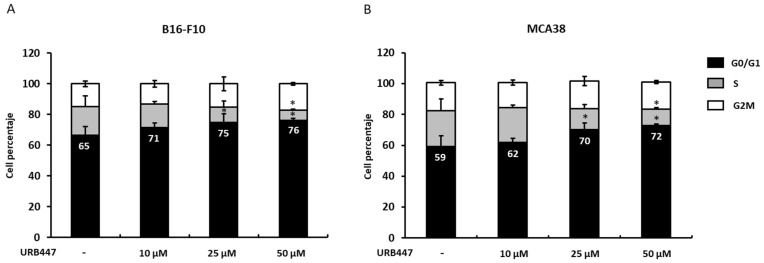
Cell cycle analysis in cells treated with URB447. Melanoma (**A**) and CRC cancer cells (**B**) were incubated in the presence of different concentrations of URB447 for 24 h. Propidium iodide RNAse Kit was used for flow cytometry and the number of cells in each cell cycle phase were quantified (*n* = 3). Differences were considered statistically significant for * *p* < 0.05 using Student *t* test.

Since cell cycle dysregulation is a hallmark of cancer, we aimed to analyze whether URB447 may disrupt the cell cycle and, therefore, reduce tumor cell viability, as previously observed. We reported that URB447 slightly impairs the B16 melanoma cell cycle through G0/G1 phase arrest. In detail, the percentage of cells in G0/G1 phase increased 9% when treated with 10 µM URB447 for 24 h compared to the control cells. However, 25 µM and 50 µM blocked the cell cycle in G0/G1 phase. Regarding to MCA38, no changes were detected after 24 h of treatment with 10 µM URB447. However, 25 µM and 50 µM significantly increased G0/G1 phase arrest. Both cell lines exhibited reduced cell numbers in S phase under 50 µM URB447 treatment, which further impairs the cell cycle. (Figure 4).

### 2.4. Tumor Cell Migration Is Compromised by URB447

**Figure 5:**
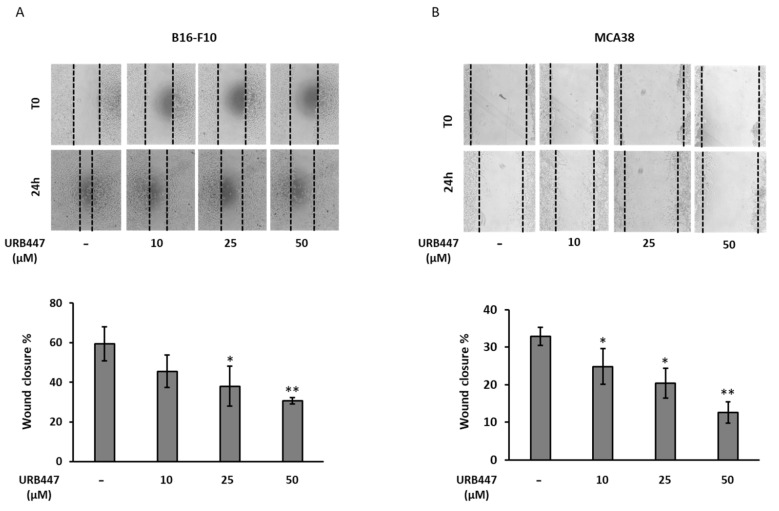
The migratory ability of tumor cells upon URB447 treatment. Wound healing assay was carried out upon stimulation of melanoma (**A**) and CRC cancer cells (**B**) in the presence of increasing concentrations of URB447 for 24 h. The closed wound area was calculated. Images show representative result (*n* = 3). Differences were considered statistically significant for * *p* < 0.05, ** *p* < 0.01 using Student *t* test.

To increase ability to step up from the primary lesion and migrate to generate distant metastasis is a required step for disease progression. To further explore the antitumor action of URB447, we carried out a wound healing assay using different URB447 concentrations. As observed in Figure 5, the highest tested concentration, 50 µM, led to a pronounced impairment of cell migration in both models. In detail, URB447 reduced 50% the migration ability in melanoma and almost 60% in colon cancer cells. Moreover, 25 µM exerted an anti-migratory effect in both cell types, decreasing the migration potential by 30%. Finally, although a reduction was observed, 10 µM did not significantly affect cell migration after 24 h in melanoma, but it reduced that of CRC in 20%.

### 2.5. Matrix Degrading MMP-2 and MMP-9 Secretion Is Not Altered by URB447

**Figure 6:**
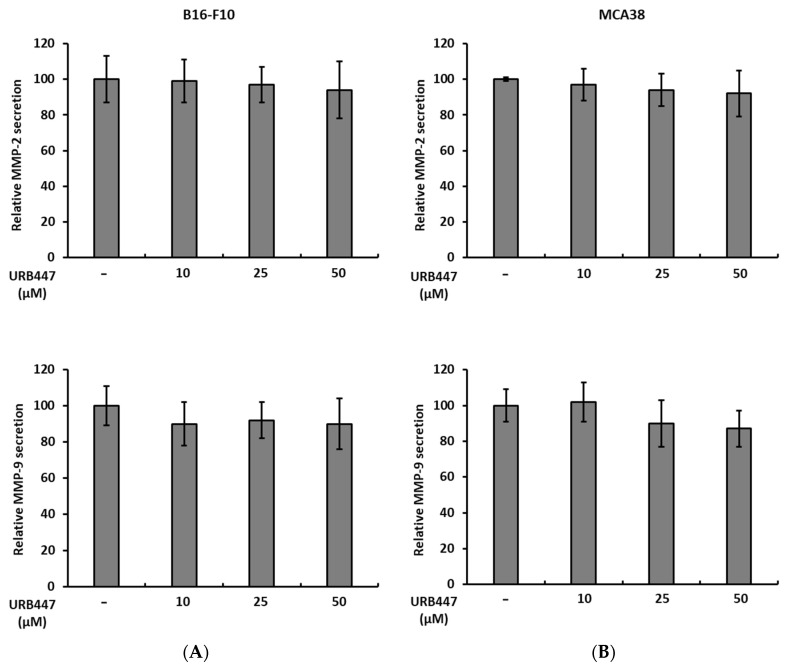
Secretion of MMP-2 and MMP-9 in URB447 treated tumor cells. The secretion of MMP-2 was measured in melanoma (**A**) and CRC cancer cells (**B**) in the presence of increasing concentrations of URB447 for 24 h. (*n* = 3). No significant differences were observed.

One of the main features of metastatic cancer cell is the ability to degrade the ECM present in both the primary tissue and the secondary organ upon metastatic colonization. Here, we show that even though URB447 slightly influences the secretion of MMP-2 and MMP-9 in both tumor models, there is no significant change after 24 h. Although a reduction trend can be observed, URB447 did not disturb the secretion of matrix degrading proteins (Figure 6). 

### 2.6. Daily Treatment with URB447 Reduced the Metastatic Burden in the Liver

**Figure 7:**
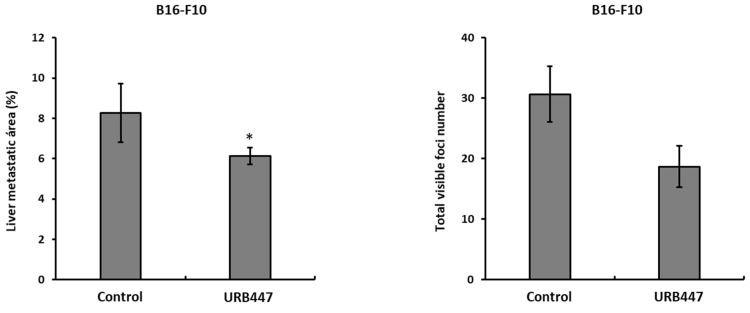
Metastatic growth of melanoma in control and URB447 treated mice liver. Mice were intrasplenically injected with 2 × 10^5^ B16-F10 melanoma cells and treated with Vehicle or URB447 1 mg/kg for 10 days. The metastatic area and visible foci number were quantified. (*n* = 4). Differences were considered statistically significant for * *p* < 0.05 using Student *t* test.

The obtained results may uncover a beneficial effect of URB447 during liver colonization and tumor growth. Liver metastasis orthotopic model revealed that daily treatment of tumor bearing mice with URB447 led to reduced tumor burden in the liver in a melanoma model. In detail, the metastatic area was reduced in 25% in melanoma compared to vehicle treated group, after a daily i.p. injection of 1 mg/kg URB447 (Figure 7). Moreover, the number of visible melanoma foci in the URB447 treated group was reduced, although the differences were not statistically significant.

## 3. Discussion

Cannabinoid receptors are gaining relevance not only in nervous system linked pathologies, but also in a wide array of pathologies with different origins [22,23,24]. Among others, their role during cancer progression has been uncovered, pointing out cannabinoids as a potential target for disease management. Here, we show that a CB2 receptor agonist synthetic cannabinoid with no psychoactive effects, URB447, reduces several prometastatic properties of cancer cells. Finally, daily treatment with URB447 affects tumor features of melanoma, leading to reduced metastatic growth in the liver.

We have reported a dose-dependent reduction in melanoma and CRC cell viability when exposed to URB447. These findings are in line with recent studies using a new CB2 agonist reporting the same trend [25]. We observed that this decreased cell viability is partly mediated by tumor cell apoptosis. Previous studies from other groups have linked CB2 receptor agonists with this phenomenon, pointing out to Caspase 3 and 7 increase as responsible for the observed phenotype, along with PARP expression stimulation [26], which may account for the reported observations. Moreover, CB2 stimulation leads to antiapoptotic protein Bcl-2 reduction [27], therefore, facilitating cell death. A second mechanism that seems to be involved in cancer cell reduced viability. URB447 treatment altered cell cycle in both models, leading to G0/G1 cell cycle arrest, accompanied with decrease in S phase cell counts. This finding goes along a recent study reporting G1 phase arrest in CB2 stimulated CRC cancer cells. Furthermore, they found reduced levels of cyclins in CB2 stimulated colorectal CRC cells [26].Moreover, CDK4 expression was also reduced when agonizing a CB2 receptor in a glioblastoma cancer model [28], which may explain the results obtained using URB447. 

The aggressiveness of cancer cells increases when they undergo EMT and, therefore, acquire increased motility to leave the primary lesion and colonize distant organs. Interestingly, URB447 impaired cancer cell migration in a dose-dependent trend. Thus, the involvement of CB2 receptor in the regulation of tumor cell migration needs further attention. In this regard, the partial CB2 receptor agonist, CBD, showed anti-migratory effects on pancreatic cancer [29], in line with our observations. Interestingly, CBD blocked epithelial growth factor mediated lung cancer cell migration in vitro controlling the levels of EMT genes, such as vimentin [30], supporting our observations. Moreover, the CB2 receptor agonist reduced MMP-9 secretion in dendritic cells [31], impairing their migration, which may also occur in cancer cells. Regarding the expression of MMPs, URB447 did not alter the secretion of metalloproteases after 24 h incubation. However, serum starvation of tumor cells could help elucidate the effect of URB447 in MMP secretion, since 1% serum exhibited MMP activity, which may uncover the differences generated by URB447.

In vivo, URB447 reduced the metastatic burden in a melanoma model of liver metastasis. In detail, daily treatment with 1 mg/kg URB447 impaired metastatic growth in the liver in 25% in melanoma. The reported reduction in cell viability and cell cycle arrest might partially mediate the observed effect in vivo. Moreover, proapoptotic action of URB447 might help reduce liver metastatic area. Apart from the direct effect of URB447 in tumor cells, a potential action in the colonized organ must be taken into account. In this regard, the synthetic atypical cannabinoid Abn-CBD, a cannabidiol (CBD) derivative is effective in reducing liver related inflammation and subsequent liver damage during non-alcoholic fatty liver disease [32]. In line with this report, a selective CB2 agonist protected the liver from bile duct ligation mediated injury through the impairment of the inflammatory response [33]. Similarly, the inhibition of the inflammatory response is involved in the CB2 receptor mediated liver protection during alcohol-related injury [34]. Regarding the non-parenchymal cells in the liver, in vivo knock out of the CB2 receptor drove the augmented inflammatory response of hepatic stellate cells and increased liver damage, worsening the CCl4 promoted liver fibrosis [35]. Therefore, URB447 might reduce the inflammatory and fibrotic status of the liver, impairing immune suppression and tumor-associated collagen accumulation, thus, slowing down tumor growth. 

Further studies will unravel the role of liver non-parenchymal cells upon URB447 treatment and the regulation of the immune response under the CB2-stimulated microenvironment.

## 4. Materials and Methods

### 4.1. Animals

C57BL/6J male mice (6–8 weeks old) were obtained from Charles River (Barcelona, Spain). Institutional guidelines and national laws for experimental care guidelines were followed for animal housing, care and experimental conditions. Animal conditions were fulfilled with unlimited food and water availability. All the conducted in vivo experiments were approved by the Basque Country University Ethical Committee (CEID) and by institutional, national and international guidelines for animals use in research activities.

### 4.2. Cell Lines and Reagents

The mouse malignant melanoma B16-F10 and colon carcinoma MCA38, both syngeneic with C57BL/6J mice were obtained from the American Type Culture Collection (ATCC, LGC Standards SLU). MCA38 cells were cultured in RPMI-1640 medium and B16-F10 cells were maintained in DMEM medium. To get the complete medium, media were supplemented with heat-inactivated 10% fetal bovine serum (FBS), penicillin (100 U/mL), streptomycin (100 µg/mL) and amphotericin B (0.25 µg/mL). All the reagents were purchased from Thermo Fisher Scientific (Waltham, MA, USA). Cancer cell lines were discarded after ten passages and substituted by new batches. The synthetic cannabinoid URB447 was purchased from Cayman Chemicals (Ann Arbor, MI, USA).

### 4.3. Cell Viability

In total, 5 × 10^3^ /well cancer cells were cultured in 96-well plates and supplemented with complete growth medium for 24 h. Afterwards, different concentrations of URB447 ranging from 10 µM to 50 µM were added to cancer cell cultures in 1% FBS supplemented medium. Control cells were treated with 0.1% DMSO (final concentration) diluted in 1% FBS containing fresh medium. Treatment effectiveness was measured after 24 and 48 h, incubating tumor cells for 2 h with PrestoBlue cell viability reagent (Thermo Fisher Scientific (Waltham, MA, USA)) following manufacturer’s indications. 

### 4.4. Apoptotic Cell Determination

Apoptosis analysis was carried out by culturing 3 × 10^5^ cancer cells in 6-well plates for 18 h in complete medium. The medium was changed and cells were treated with 10, 25 and 50 µM URB447 in fresh medium supplemented with 1% FBS for 24 h (control cells were treated with 0.1% DMSO). The supernatant was collected and attached cells were trypsinized and added into the same tube as floating cells. After washing two times with PBS, Dead Cell Apoptosis Kit with Annexin V FITC and PI (Thermo Fisher Scientific (Waltham, MA, USA)) was used to quantify the apoptosis mediated by URB447, following manufacturer’s instructions. Apoptosis was evaluated by flow cytometry using the Gallios cytometer (Beckman Coulter, Brea, CA, USA).

### 4.5. Cell Cycle Analysis

For cell cycle analysis, 3 × 10^5^ cancer cells were cultured in 6-well plates for 18 h in complete medium. The medium was changed and cells were treated with 10, 25 and 50 µM URB447 in fresh medium supplemented with 1% FBS for 24 h (0.1% DMSO was used as vehicle treatment for control cells). Attached cells were collected through trypsinization and subjected to phosphate-buffered saline (PBS) washes prior to fixation using 70% Ethanol for 30 min at 4 degrees. Ethanol was eliminated from cells through PBS washes prior to cell staining using propidium iodide (PI) containing FxCycle PI/RNase Solution (Thermo Fisher Scientific (Waltham, MA, USA)) following the manufacturer’s indications. Finally, the URB447 mediated perturbation of cell cycle was evaluated by flow cytometry using the Gallios cytometer (Beckman Coulter, Brea, CA, USA).

### 4.6. Wound Healing Assay

Cancer cells were cultured at 2 × 10^5^ cells/well in 24-well plates in complete medium for 24 h. B16-F10 and MCA38 cells were incubated with 25 µg/mL and 1 µg/mL Mytomicin C, respectively (Fisher Scientific, Madrid, Spain) for 2 h. Then, a scratch was made using the 200 µL tip, followed by three washes to eliminate detached cells. Finally, control cells were incubated with 0.5% DMSO whereas treated cells were incubated with 50, 25 and 10 µM URB447 in 1% FBS supplemented medium. Pictures were taken at the time of treatment addition (T0) and after 24 h (T 24 h). The total wound area was compared between that of T0 and T24. Results are shown as the percental of wound area closed.

### 4.7. Zymography

The secretion of MMPs was determined by gelatin zymography as previously described. A total 2 × 10^5^ cells /mL were cultured for 24 h in complete medium supplemented with 10% FBS. Then, the medium was changed for 1% FBS containing medium and treatments were added for 24 h. Finally, control and URB447-treated cell supernatants were collected and centrifuged for 5 min at 4000 rpm. Supernatants were run in 1% gelatin containing 10% bis-acrylamide gels. For gelatin digestion, gels were incubated overnight in developing buffer followed by staining in Coomassie Blue solution (BioRad, Hercules, CA, USA). Digested bands were quantified using Image J2 software (National Institutes of Health, Bethesda, MD, USA).

### 4.8. In Vivo Liver Metastasis Assay

C57BL/6J male mice (6–8 weeks old) were anesthetized using Xilazyn/Ketamin solution. A thin cut was made in the left flank under the ribs to expose the spleen. Then, 2 × 10^5^ B16-F10 cells were injected in the distal pole of the spleen of each mouse diluted in 100 µL PBS (2 × 10^6^ cels/mL concentration). The spleen was carefully relocated and the wound was closed. Mice were treated with intraperitoneal injections of 1 mg/kg URB447 (200 µL/mouse) from day 1 to day 10 and sacrificed 14 days after tumor cell inoculation (3% URB447 in DMSO, 97% PBS). Control animals were treated with vehicle solution (3% DMSO in PBS). All the procedures involving animals were approved by the University of the Basque Country and the Basque Government Ethics committee (ethical approval code M20_2020_315).

## Data Availability

Data is contained within the article.
